# Scleroderma and dentistry: Two case reports

**DOI:** 10.1186/s13256-016-1086-1

**Published:** 2016-10-24

**Authors:** Shantanu Dixit, Chaithra Kalkur, Atul P. Sattur, Michael M. Bornstein, Fred Melton

**Affiliations:** 1 Department of Oral Medicine and Radiology, Dhulikhel hospital, Kathmandu University School of Medical Sciences, Dhulikhel, Nepal; 2 Department of Oral Medicine and Radiology, Century International Institute of Dental Science and Research Centre, Kasargod, Kerala India; 3 Department of Oral Medicine and Radiology, SDM College of Dental Sciences, Sattur, Karnataka India; 4 Department of Oral Surgery and Stomatology, School of Dental Medicine, University of Bern, Bern, Switzerland; 5 General Practitioner, Wenatchee, Washington USA

**Keywords:** CREST syndrome, Localized scleroderma, Morphea, Parry–Romberg syndrome, Raynaud’s phenomenon, Systemic sclerosis

## Abstract

**Background:**

Scleroderma is a chronic connective tissue disorder with unknown etiology. It is characterized by excessive deposition of extracellular matrix in the connective tissues causing vascular disturbances which can result in tissue hypoxia. These changes are manifested as atrophy of the skin and/or mucosa, subcutaneous tissue, muscles, and internal organs. Such changes can be classified into two types, namely, morphea (localized) and diffuse (systemic). Morphea can manifest itself as hemifacial atrophy (Parry–Romberg syndrome) although this remains debatable. Hence, we present a case of morphea, associated with Parry–Romberg syndrome, and a second case with the classical signs of progressive systemic sclerosis.

**Case presentation:**

Case one: A 20-year-old man of Dravidian origin presented to our out-patient department with a complaint of facial asymmetry, difficulty in speech, and loss of taste sensation over the last 2 years. There was no history of facial trauma. After physical and radiological investigations, we found gross asymmetry of the left side of his face, a scar on his chin, tongue atrophy, relative microdontia, thinning of the ramus/body of his mandible, and sclerotic lesions on his trunk. Serological investigations were positive for antinuclear antibody for double-stranded deoxyribonucleic acid and mitochondria. A biopsy was suggestive of morphea. Hence, our final diagnosis was mixed morphea with Parry–Romberg syndrome.

Case two: A 53-year-old woman ﻿of Dravidian origin presented to our out-patient department with a complaint of gradually decreasing mouth opening over the past 7 years. Her medical history was noncontributory. On clinical examination, we found her perioral, neck, and hand skin to be sclerotic. Also, her fingers exhibited bilateral telangiectasia. An oral examination revealed completely edentulous arches as well as xerostomia and candidiasis. Her serological reports were positive for antinuclear antibodies against centromere B, Scl-70, and Ro-52. A hand and wrist radiograph revealed acro-osteolysis of the middle finger on her right hand. Hence, our final diagnosis was progressive systemic sclerosis.

**Conclusion:**

Through this article, we have tried to emphasize the importance of a general examination when diagnosing rare systemic diseases such as scleroderma and the role of the general dentist when caring for such patients, even though they can be quite rare in general practice.

## Background

Scleroderma is a chronic sclerosing disease of the connective tissues. It derives its name from the Greek words *scleros* (hard) and *derma* (skin). Hidebound skin is an important and characteristic feature of this disease thereby making the name “hidebound disease” more popular [1, 2]. The first description of this disease as a separate pathological entity was given by Carlo Curzio of Naples in 1752 [2, 3] and its first name, “sclerodermie,” was given nearly a century later by Gintrac in 1847. For years, the disease was thought to be a dermatological disorder but after its distinctive characteristic of systemic involvement was proven, Goetz coined the term “progressive systemic sclerosis” in 1945 [2, 4].

Scleroderma exists in two forms: morphea (circumscribed scleroderma) and generalized/progressive (diffuse scleroderma). However, some articles have mentioned acrosclerosis (scleroderma of the peripheries associated with Raynaud’s phenomenon) as a third form [5, 6]. Morphea (localized scleroderma) is again subdivided into five types, namely: plaque, generalized, bullous, linear, and deep [7, 8]. CREST syndrome (calcinosis cutis, Raynaud’s phenomenon, esophageal dysfunction, sclerodactyly, and telangiectasia), a rare condition, is thought to be a heterogeneous variant of systemic scleroderma [9]. Similarly, Parry–Romberg syndrome (hemifacial atrophy) is thought to be another variant of scleroderma. However, this remains debatable [8, 10, 11].

Scleroderma was described as a pathological entity in the mid-eighteenth century but to date the exact etiopathogenesis is still unknown, possibly because of the disease’s rarity. However, it is understood that age, gender, genetic, and environmental factors can influence vulnerability for the disease [2]. Some authors have suggested viral or bacterial infection (*Borrelia burgdorferi*), particularly in the case of morphea, as the causative agent. But sufficient evidence to support such claims is lacking [8]. Untimely interaction of immunity with the vascular system resulting in endothelial damage is also hypothesized as a major contributor for the initiation and progression of the disease [2, 12].

Systemic sclerosis (diffuse/generalized/progressive scleroderma) initially manifests itself as pitting edema of the skin which progresses to a thickening and hardening of the skin. The systemic form can involve multiple organs, such as the kidneys, lungs, heart, and the gastrointestinal system, among which the involvement of the latter is most common. These organs are affected either by fibrosis or by a diminished blood supply. Raynaud’s phenomenon (paroxysmal vasospasm), claw-like fingers, hyperpigmentation, telangiectasia, and subcutaneous calcification are other common systemic manifestations that can be seen. Oral and facial tissues are the most affected, manifesting as the characteristic mask-like face (Mona Lisa face), constricted lips (fish mouth), a narrowing and stiffening of the tongue (chicken tongue), microstomia, pseudoankylosis, resorption of the mandibular angle, xerostomia, dental caries, and a widening, radiographically, of periodontal ligaments [2, 13]. Oral ulceration, secondary to gastroesophageal reflux disease (GERD), is also reported in some cases [6]. Most of the time, as a result of these manifestations, patients visit dentists for these aesthetical and facial dysfunctions.

Morphea (localized scleroderma), on the other hand, causes localized fibrosis similar to scleroderma, resulting in localized indurated lesions [14]. As mentioned earlier, morphea can be subdivided into five different types. Mixed forms, such as localized scleroderma of the face with plaque/linear morphea at some other site, have been reported in the literature [15].

The face is the most common site for the linear type of morphea with fibrosed tissue appearing as a sabre cut (*en coup de sabre*) resulting in facial hemiatrophy mimicking Parry–Romberg syndrome [2]. Hence, with this article we are presenting two such cases of scleroderma reporting to us with aesthetical and facial dysfunctions. Both cases were later diagnosed as mixed morphea, with Parry–Romberg syndrome, and progressive systemic sclerosis, respectively.

## Case presentation

### Case one

A 20-year-old man of Dravidian origin presented to our Department of Oral Medicine and Radiology with the chief complaint of facial asymmetry over the past 2 years. According to him, and on review of photographs he had brought, his facial symmetry was within normal limits prior to these past 2 years. He also reported difficulty in articulation and a loss of taste sensation. He also presented with skin lesions on his trunk (abdomen and back).

**Fig. 1:**
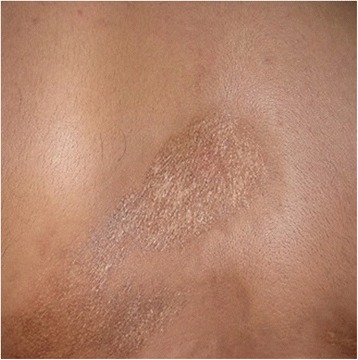
Morphea seen on trunk (back) of patient

**Fig. 2:**
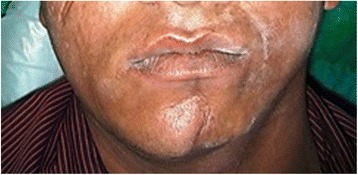
Gross asymmetry of face (left side) with “*en coup de sabre*” on chin

His medical and family histories were noncontributory. On general examination, he was found to be conscious and oriented. His vital signs were within normal range. He had limited, well-circumscribed, and oval-shaped hyperpigmented lesions on his abdomen and back (Fig. 1). These lesions appeared fibrotic on palpation. A head and neck examination revealed gross asymmetry of the left side of his face and there was a sharp demarcation on his chin, separating the normal and abnormal side (Fig. 2).

**Fig. 3:**
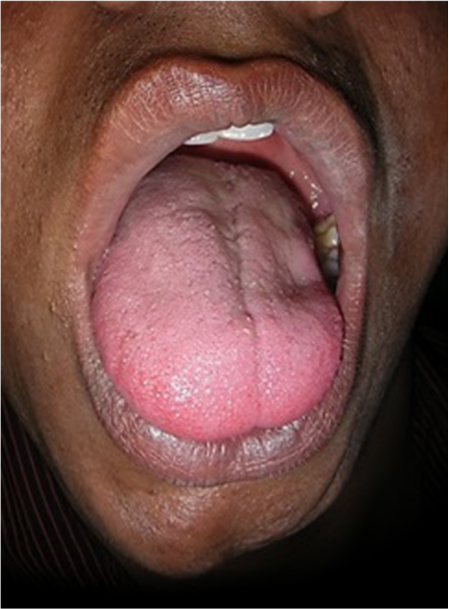
Atrophy of tongue (left side)

A visually evident loss of fat, muscles, and subcutaneous tissue resulted in a shrunken appearance to the left side of his face. His left ear pinna appeared to be fibrosed on palpation. Skin on the affected side appeared to be sclerotic. An ocular examination disclosed no abnormalities. An intraoral examination revealed shrunken and rigid left side of his tongue otherwise his mucosa appeared to be normally moist (Fig. 3). A hard tissue examination offered no relevant findings.

Hence, our provisional diagnosis was facial hemiatrophy with plaque morphea and further investigation was recommended.

**Fig. 4:**
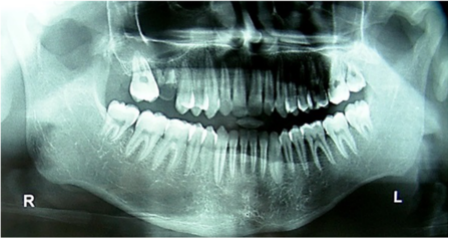
Panoramic view showing gross asymmetry of face (left side) with relative microdontia and thinning of ramus/body of mandible

Panoramic view showed relative microdontia on the left side, with a thinning of his mandibular body and ramus (Fig. 4). Submentovertex and posteroanterior views also revealed gross asymmetry of the left side of his jaw. Serological investigations were positive for double-stranded deoxyribonucleic acid (DNA) and antimitochondrial antibody; however, his erythrocyte sedimentation rate (ESR) levels were normal.

Fundoscopic examination of his eyes and audiometry showed no abnormalities. Ultrasonography examinations of his bilateral buccal mucosa and parotid glands were normal. A skin biopsy revealed a thickened epidermis with sparse adnexal structures. Thickened and closely packed collagen bundles in reticular dermis, along with hypocellularity and sparse lymphoplasmacytic infiltrate in the dermis, were suggestive of morphea.

After correlating these findings with the available literature, the final diagnosis of mixed morphea with Parry–Romberg syndrome was given.

### Case two

**Fig. 5:**
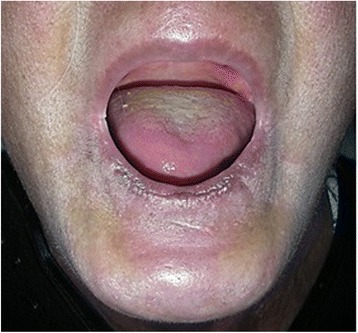
Microstomia resulting in difficult prosthesis placement

**Fig. 6:**
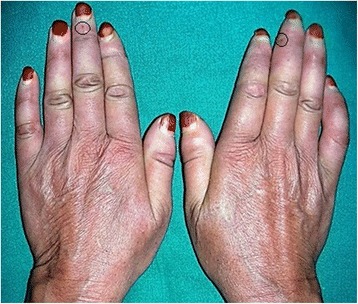
Shiny and tense skin of hand showing Raynaud’s phenomenon and telangiectasia (*encircled*)

A 53-year-old woman of Dravidian origin visited our department complaining of difficulty in using her complete dentures as a result of the gradual reduction in mouth opening over the previous 7 years. She also complained of a burning sensation of her oral mucosa (predominantly in her palate) and a tightness of the skin in her perioral and neck regions. On clinical examination we found her face to be expressionless. On palpation, the skin of her perioral and neck regions was sclerotic (Fig. 5). The skin on her fingers also appeared to be pale on inspection and indurated when palpated. According to our patient, this paleness spreads symmetrically to all ten fingers when exposed to cold, suggestive of Raynaud’s phenomenon. Her fingers also showed areas of telangiectasia (Fig. 6). An oral examination revealed completely edentulous maxillary and mandibular arches, microstomia due to rigid perioral skin, xerostomia (positive tongue blade test), and an erythematous patch on her palate. The clinical findings were suggestive of progressive systemic sclerosis; hence, we recommended radiological and serological investigations. For the erythematous lesion, a swab was taken and was sent for culture. These investigative reports were consistent with our provisional diagnosis.

**Fig. 7:**
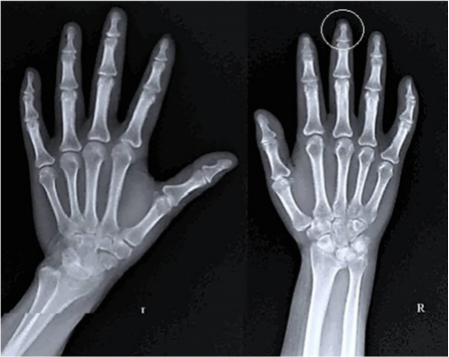
Hand and Wrist radiograph revealing acro-osteolysis (encircled) of terminal phalanges of right middle finger

The serological reports were positive for increased serum C3 level and a ribonucleic acid (RNA) profile was positive for centromere B, Scl-70, and Ro-52, explaining her inability to use her prostheses with which she had been comfortable 7 years prior. In addition, a radiograph of her hand and wrist revealed reabsorption of the terminal phalanges (acro-osteolysis) of the middle finger on her right hand (Fig. 7). A swab culture was positive for candidiasis, which reflected alteration of her oral microflora secondary to xerostomia, resulting in a burning sensation.

## Discussion

Progressive/diffuse/generalized sclerosis is more common in the fourth to sixth decades of life and it affects females more than males with a 3 to 6:1 ratio, respectively [16]. The American College of Rheumatology has recommended criteria for the diagnosis of systemic scleroderma with the major criterion as proximal diffuse (truncal) sclerosis (skin tightness, thickening, non-pitting induration). Minor criteria include: sclerodactyly (only fingers and/or toes), digital pitting scars or loss of substance of the digital finger pads (pulp loss), and bibasilar pulmonary fibrosis.

Our second case fulfils the major criteria of symmetrical thickening of the skin proximal to the metacarpophalangeal joint, as shown in Fig. 6. Our patient also exhibited Raynaud’s phenomenon, along with other cardinal features, which are the most common and earliest manifestations of the disease in 95 % of cases, as suggested by Lester *et al*. [14] and other authors [6]. Localized scleroderma/morphea is more common in white women with a female to male ratio of 2 to 4:1 and an incidence rate of 0.3 to 3 cases per 100,000 individuals/year.

Morphea (localized scleroderma) causes localized fibrosis without systemic involvement and exists in five different forms but also can persist in “mixed form” where localized scleroderma of the face can be associated with plaque morphea or linear scleroderma affecting other parts of the body, most often the trunk. Linear scleroderma, a subtype of morphea, is strongly associated with Parry–Romberg syndrome (facial hemiatrophy); however, the relationship is debatable. Linear scleroderma is characterized by unilateral atrophies of the skin, subcutaneous tissues, muscles, and underlying bone structures with neurological deficits [8]. Authors have mentioned the coexistence of *en coup de sabre* or linear scleroderma with Parry–Romberg syndrome in 42 % of cases [17]. Tollefson and Witman [18] have also reported this overlapping of the two different diseases in 36.6 % of patients. Our second case was an illustration of the coincidence of these two diseases. Differential diagnosis of scleroderma on the basis of vascular changes can be primary Raynaud’s phenomenon, physical trauma, chemical exposure, and drugs and/or toxins. On the basis of skin changes, amyloidosis, insulin-dependent diabetes mellitus, metabolic-genetic disorder, and overlap syndrome can be the differential diagnoses. Ageing, sarcoidosis, amyloidosis, and autoimmune connective tissue disease can be considered in the differential diagnosis in cases of visceral involvement [2].

There are different autoantibodies which are extremely helpful in forecasting different subtypes of scleroderma. However, autoantibody absence does not preclude the diagnosis of this disease as 20 % of the patients with different subtypes of scleroderma do not show these antibodies. Highly specific antinuclear antibodies (ANA) for scleroderma include anti-single-stranded, anti-histone, and anti-topoisomerase antibodies. Some other ANA that commonly present in scleroderma include anticentromere, anti-U3-RNP, anti-Th, anti-fibrillin, antiphospholipid, and antimitochondrial antibodies [19–21]. Both of our cases were positive for a few of these serological markers and the markers were extremely helpful in yielding a final diagnosis.

Treatment of these types of cases is vastly difficult to plan as the exact etiology for this disease is not exactly known. Medical treatments include topical, intralesional, or systemic glucocorticoids, vitamin E, Vitamin D3, retinoid, penicillin, griseofulvin, and interferon-alpha. For those with a more severe and progressive disease, treatment includes methotrexate (for which there is limited evidence in linear scleroderma), corticosteroids, cyclophosphamide, and azathioprine, although it remains unclear as to how beneficial such treatment modalities are. Surgical intervention includes restorative plastic surgery (fat or silicone implants, flap/pedicle grafts, or bone implants) [22, 23].

Preservation or replacement of the existing dentition is a great challenge for a dentist in these cases. Patients should be instructed regarding the importance of maintaining proper oral hygiene and counseled frequently in order to prevent the patient undergoing depression due to the challenging nature of the disease. Mouth dilator exercises such as increasing the number of tongue blades/ice cream sticks between the posterior teeth, should be encouraged. Difficulty in holding a toothbrush secondary to sclerodactyly can be overcome by advising patients to use adaptive devices such as electric toothbrushes, Waterpik flossers, and floss forks. Temporomandibular/myofacial pain dysfunction can be treated with the use of muscle relaxants, physiotherapy, and dental appliances. Dysgeusia and dental caries secondary to xerostomia can be avoided by advising the use of artificial saliva, sugar free candies, fluoride toothpaste and medications like pilocarpine [2]. In severe cases, surgical procedures, such as commissurotomy, are recommended to increase mouth opening [24].

## Conclusions

With these two case reports we have tried to elucidate the effects of systemic and localized scleroderma in the maxillofacial region, as well as discuss systemic manifestations. Appropriate management of scleroderma requires frequent consultations with a rheumatologist but general dentists should monitor patients with scleroderma by performing frequent clinical and radiological examinations so as to follow the course of the disease and to meet its dental challenges.

## References

[1] Ahathya RS, Deepa Lakshmi D, Emmadi P. Systemic sclerosis. Indian J Dent Res. 2007;18:27–30. 10.4103/0970-9290.30919 17347542

[2] Vikram B, et al. A rare case of hidebound disease with dental implications. Dent Res J (Isfahan). 2013;10(4):556–61. PMC3793424 24130596

[3] Naylor WP. Oral management of scleroderma patient. J Am Dent Assoc. 1982;105:814–7. 10.14219/jada.archive.1982.0466 6960074

[4] Goetz RH. The pathology of progressive systemic sclerosis with special reference to changes in the viscera. Clin Proc. 1945;4:337–92.

[5] Alexandridis C, White SC. Periodontal ligament changes in patients with progressive systemic sclerosis. Oral Surg Oral Med Oral Path. 1984;58:113–8. 10.1016/0030-4220(84)90375-x 6589569

[6] Anbiaee N, Tafakhori Z. Early diagnosis of progressive systemic sclerosis (scleroderma) from a panoramic view: report of three cases. Dentomaxillofac Radiol. 2011;40(7):457–62. 10.1259/dmfr/64340754 PMC3528135 21960405

[7] Peterson LS, et al. Classification of morphea (Localized Scleroderma). Mayo Clin Proc. 1995;70:1068–76. 10.4065/70.11.1068 7475336

[8] Careta MF, Romiti R. Localized scleroderma: clinical spectrum and therapeutic update. An Bras Dermatol. 2015;90(1):62–73. 10.1590/abd1806-4841.20152890 PMC4323700 25672301

[9] Neville BW, Damm DD, Allen CM, Bouquot JE. Oral and maxillofacial pathology. 3rd ed. St Louis: Elsevier/Saunders; 2009. p. 799.

[10] Gambichler T, et al. Bilateral linear scleroderma “*En coup de sabre*” associated with facial atrophy and neurological complications. BMC Dermatol. 2001;1:9. 10.1186/1471-5945-1-9 PMC61032 11741509

[11] Vierra E, Cunningham BB. Morphea and localized scleroderma in children. Semin Cutan Med Surg. 1999;18:210–25. 10.1016/s1085-5629(99)80019-4 10468041

[12] Lever WF, Schaumburg-Lever G. Histopathology of the Skin. 6th ed. Philadelphia: JB Lippincott Co; 1983. p. 464–6.

[13] Caplan HI, Benny RA. Total osteolysis of the mandibular condyle in progressive systemic sclerosis. Oral Surg Oral Med Oral Path. 1978;46:362–6. 10.1016/0030-4220(78)90400-0 278933

[14] Lester WB, Martin SG, Michael G, Jonathan AS. In: Greenberg M, Glick M, Ship JA, editors. Burket's oral medicine: Diagnosis and treatment. Immunologic diseases. 11th ed. New York: JB Lippincott Publishing Co. Ltd; 2008. p. 451.

[15] Marzano AV, et al. Localized scleroderma in adults and children. Clinical and laboratory investigations on 239 cases. Eur J Dermatol. 2003;13:171–6. 12695134

[16] Cawson RA, Odell EW. Cawson’s essentials of oral pathology and oral medicine. 8th ed. London: Churchill Livingstone; 2008. p. 195.

[17] Sommer A, et al. Clinical and serological characteristics of progressive facial hemiatrophy: a case series of 12 patients. J Am Acad Dermatol. 2006;54:227–33. 10.1016/j.jaad.2005.10.020 16443052

[18] Tollefson MM, Witman PM. *En coup de sabre* morphea and Parry-Romberg syndrome: a retrospective review of 54 patients. J Am Acad Dermatol. 2007;56:257–63. 10.1016/j.jaad.2006.10.959 17147965

[19] Takehara K, Sato S. Localized scleroderma is an autoimmune disorder. Rheumatology. 2005;44:274–9. 10.1093/rheumatology/keh487 15561734

[20] McPhee SJ, Papadakis M, Rabow MW. Current Medical Diagnosis and Treatment. 51st ed. USA: McGraw-Hill companies, Inc; 2012. p. 815–7.

[21] Chung L, Utz PJ. Antibodies in Scleroderma: Direct Pathogenicity and Phenotypic Associations. Curr Rheumatol Rep. 2004;6(2):156–63. 10.1007/s11926-004-0061-9 15016347

[22] Hunzelmann N, et al. Management of localized scleroderma. Semin Cutan Med Surg. 1998;17:31–4. 10.1016/s1085-5629(98)80060-6 9512105

[23] Stone J. Parry-Romberg syndrome. Pract Neurol. 2006;6:185–8.

[24] Johns FR, Sandler NA, Ochs MW. The use of a triangular pedicle flap for oral commissuroplasty: Report of a case. J Oral Maxillofac Surg. 1998;56:228–31. 10.1016/s0278-2391(98)90874-6 9461150

